# Inhibition of breast cancer cell proliferation and tumorigenesis by long non-coding RNA *RPPH1* down-regulation of miR-122 expression

**DOI:** 10.1186/s12935-017-0480-0

**Published:** 2017-11-21

**Authors:** Yi Zhang, Lili Tang

**Affiliations:** Department of Breast Surgery, Xiangya Hospital Central South University, No. 87 Xiangya Road, Changsha, 410008 China

**Keywords:** lncRNA *RPPH1*, miR-122, Breast cancer, Targeted regulation, Cell proliferation

## Abstract

**Background:**

Recent studies showed that long non-coding RNA (lncRNA) plays an important role in many life activities. *RPPH1* is one of the lncRNA genes that are expressed differently between breast cancer and normal tissues by the lncRNA gene chip. Our study was conducted to examine the regulation of lncRNA *RPPH1* in breast cancer.

**Methods:**

Two cell lines, MCF-7 and MDA-MB-231, were selected to be the research objects in this study; *RPPH1* overexpression and knockdown models were established by transforming vectors. Real-time polymerase chain reaction, MTT assay, clone formation and cell flow cytometer assay were used to test the function of *RPPH1*. Dual-luciferase assay was used to detect a target relationship between *RPPH1* and miR-122.

**Results:**

*RPPH1* overexpression promoted cell cycle and proliferation and increased colony formation. In the *RPPH1* overexpression model, there was a target relationship between *RPPH1* and miR-122, and some of the downstream genes of miR-122, including *ADAM10, PKM2, NOD2* and *IGF1R*, were increased. Moreover, we found that lentivirus-mediated interference of lncRNA *RPPH1* inhibited tumour growth in nude mice.

**Conclusion:**

Breast cancer progression can be promoted by directly targeting miR-122 through lncRNA *RPPH1*. This study provided evidence that can serve as the molecular basis for improving treatment options for patients.

## Background

Breast cancer occurs in mammary epithelial tissues of malignant tumours and is one of the world’s three most commonly diagnosed cancers [1]. According to the World Cancer Research Fund International 2012 report, the United States, China and India share almost one-third of the burden of disease, accounting for approximately 25% of all new cancer cases diagnosed. By 2021, the incidence rate of breast cancer in women aged 55–69 years in China is estimated to increase from less than 6/10,000 to 1/1000, reaching a total of 250,000 cases [2]; in such situation, breast cancer can become the most common threat to women’s physical and mental health. Therefore, studies on the prevention and cure of breast cancer are of great significance in our country.

Long non-coding RNA (lncRNA), which measures more than 200 bp functional non-encoding RNAs in length [3], was once considered in the evolutionary process to be non-functional genome accumulation of ‘junk sequences’. However, in-depth research in recent years found that lncRNA plays an important role in dosage compensation, epigenetic regulation [4] and regulation of cell cycle and differentiation [5]. At present, some lncRNA genes, such as *LOC554202* [6], *BCAR4* [7], *MALAT1* [8] and *GAS5* [9] have been reported to play an important role in the occurrence and development of breast cancer.

Ribonuclease P RNA component H1 (*RPPH1*) is the RNA component of the RNase P ribonucleoprotein, an endoribonuclease that cleaves tRNA precursor molecules to form the mature 5′ termini of the tRNA sequences [10]. *RPPH1* has also been used as an internal control for RNA quantification [11–13]. Recent deep sequencing studies showed that *RPPH1* was upregulated in human gastric cancer tissues [14] and in the neocortex of patients with seizures [15]. A biochemical study has shown that RNase P takes part in the maturation lncRNA *MALAT1* [16]. Although these data suggested that *RPPH1* may be involved in disease progression in animals and humans, the regulatory mechanisms of *RPPH1* expression remain largely unknown. In this study, we intended to explore the interactions of *RPPH1* in breast cancer.

## Methods

### Cell lines, cell culture and tissue collection

The breast cancer cell lines MDA-MB-231, HCC-1937, MDA-MB-453 and MCF-7 were purchased from the Shanghai Cell Bank of the Chinese Academy of Science. The cells were cultured in RPMI 1640 medium (HyClone, Hudson, NH, USA) supplemented with 10% fetal bovine serum at 37 °C and with 5% CO_2_. Twenty paired breast tissues from patients were collected by surgical resection at Xiangya Hospital between May 2010 and November 2014; the diagnosis of breast cancer was confirmed by histopathologic evaluation. The study was approved by the ethics committee of the Xiangya Hospital of Central South University.

### RNA extraction and quantitative real-time polymerase chain reaction (qPCR)

Total RNA was extracted from the cell lines and tissues using Trizol reagent (Dongshen biotech, Guangzhou, China). Revert Aid First Strand cDNA Synthesis Kit (Thermo Scientific Fermentas, Waltham, MA, USA) was utilised for reverse transcription. Real-time PCR was performed using an ABI PRISM7300 Sequence Detection System (Applied Biosystems) with SYBR Green PCR mixture (Dongshen biotech). The sets of primers are shown in Table 1. The relative expression level was determined using the 2^−ΔΔCt^ analysis method, where *β*-actin and U6 were used as the internal standard. All reactions were run in triplicate and all experiments were carried out in three independent times.

**Table 1:** The primers used for qPCR assay

Gene name		Sequence: 5′ → 3′
RPPH1	Forward	CGA GCT GAG TGC GTC CTG TC
	Reverse	TCG CTG GCC GTG AGT CTG T
β-actin	Forward	AGG GGC CGG ACT CGT CAT ACT
	Reverse	GGC GGC ACC ACC ATG TAC CCT
ADAM10	Forward	GAA CAG AGT GCA CAC CAG GA
	Reverse	TGG CCA GAT TCA ACA AAA CAGT
Bcl-w	Forward	CAC AAG TGC AGG AGT GGA TGG
	Reverse	CCC GTA TAG AGC TGT GAA CTC C
VEGFC	Forward	CGA GGG CCT GGA GTG TG
	Reverse	CCG CAT AAT CTG CAT GGT GAT
PKM2	Forward	ACT CGG GCT GAA GGC AGT GA
	Reverse	TGT GGG GTC GCT GGT AAT GG
NOD2	Forward	CAG CCT CCG CAA GCA CTT CCA CT
	Reverse	CTC CAC GCC AAT GTC ACC CAC AG
IGF1R	Forward	GGA CAG GTC AGA GGG TTT C
	Reverse	CTC GTA ACT CTT CTC TGT GCC
NDRG3	Forward	GAG ATC ACC CAG CAC TTT GC
	Reverse	AAG CTC CAG CTC CAA CTC CA
miR-122		Purchased from GeneCipoiea, HmiRQP0056
U6		Purchased from GeneCipoiea, HmiRQP9001

### In-situ hybridisation assay (ISH)

The breast tissue chip containing breast carcinoma tissues and normal tissues was purchased from Auragene (Changsha, China). The sequence of the lncRNA *RPPH1* probe was purchased from BGI Tech company (Shenzhen, China). A hybridisation probe mixture (1:500) was added, and the operation followed the instructions of the in-situ Hybridisation Kit (Auragene) manufacturer. Finally, the stained chip was observed under optical microscope (Optec, ChongQin, China).

### Vector construction and transfection

To construct the pRNAT-U6.1/Neo-sh*RPPH1*, which served as the *RPPH1* knockdown vector, an *RPPH1* shRNA fragment that contained the target gene, a loop ring and the *Bam*HI and *Hin*dIII sites was inserted into the pRNAT-U6.1/Neo vector. The target sequence of the *RPPH1* shRNA was 5′-AAGTGAGTTCAATGGCTGAGG-3′. To construct the pcDNA3.1-oe *RPPH1* vector, which was the *RPPH1* overexpression vector, the target gene fragment was cloned into the site between the *Hin*dIII//*Xho*I of the pcDNA3.1 vector. Construction of both vectors with the target gene and correct insertion without nucleotide mutation or non-specific bands were confirmed.

For transfection of the *RPPH1* knockdown and overexpression vectors, MCF-7 and MDA-MB-231 cells were seeded into a 60-mm dish at 37 °C and with 5% CO_2_ until the confluence of the cells was about 50–60%. The 2.5 μg negative control vector, *RPPH1* overexpression vector, random sequence interference plasmid and *RPPH1* knockdown vector were transfected using the lipo6000 reagent (Beyotime Biotechnology, China) for 4 h in the culture without fetal bovine serum (FBS), before transferring into the complete medium for 48 h.

### MTT assay

The cells were collected and adjusted to a concentration of 5 × 10^4^ cells/well before they were cultured in a 35-mm dishat 37 °C with 5% CO_2_ for 24, 48 and 72 h. Thereafter, 10 μL of MTT was added to the cells; the solution was maintained at 37 °C with 5% CO_2_ for another 4 h before MTT was removed. The value of optical density was measured at 570 nm while the cells were suspended in 150 μL of dimethyl sulfoxide.

### Colony formation assay

The clonogenicity of a single cell was detected by colony assay. Cells were collected by adding 0.25% trypsin and were adjusted to a concentration of 300 cells/petri dish, which was then loaded with 2 mL of pre-heated culture media before being cultured at 37 °C with 5% CO_2_ for 2–3 weeks. Colony formation was terminated until the colony was visible to the naked eye. Thereafter, the cells were washed twice with phosphate-buffered saline (PBS) and fixated with 4% paraformaldehyde for 15 min before Giemsa staining for 10 min. Thereafter, the number of clones was counted and the rate of colony formation was calculated as follows: Colony formation rate = (number of colonies/inoculation cell number) × 100.

### Cell cycle analysis

The cell cycle was measured by flow cytometer. The cells were collected by trypsin (Auragene) and were washed twice with PBS before fixation in 500 μL of 75% pre-cooled ethanol at 4 °C overnight. Thereafter, the cells were washed by PBS twice, bathed in water with 100 μL of RNase A (Auragene) at 37 °C for 30 min then incubated in the dark with 500 μL of PI (Solarbio, China) at 4 °C for 30 min. Thereafter, the stained cells were analysed in a FACScanto II (BD Biosciences, USA) and the results were recorded at an excitation wave length of 488 nm.

### Luciferase reporter assay

The possibility of miR-122 and *RPPH1* target binding was predicted using an online software miRcode (http://www.mircode.org/). The predicted *RPPH1* mRNA binding site region was cloned into a psi-CHECK2 vector. MCF-7 and MDA-MB-231 cells were co-transfected with the vectors and the miR-122 mimics or scramble control. The supernatants were collected 48 h later; luciferase activity was measured using a Dual-Luciferase Reporter Assay System (Promega, Fitchburg, WI, USA).

### Tumour formation in nude mice

To assess the tumour forming potential of the parental and transfected cell lines, the in vitro cells in the log growth phase were trypsinised, washed in a serum-free medium and quantified by coulter counter before being injected into nude mice at a concentration of 2 × 10^6^ cells. Each mouse was monitored once a week for tumour development. Sites were scored as positive for tumour growth when they reached 5 mm in diameter. Tumour volume was calculated using the formula: V (mm^3^) = 0.2618 × a × b × (a + b), where a represented the maximum longitudinal diameter and b represented the maximum transverse diameter. Four weeks later, the mice were sacrificed. The entire animal study was approved by the ethics committee of the Xiangya Hospital of Central South University.

### Statistical analysis

Statistical analysis was performed using the GraphPad Prism 6 software (GraphPad Software, Inc., La Jolla, CA, USA) or SPSS 18.0 software (SPSS, Chicago, IL, USA). Data were shown as mean ± standard deviation and were analysed using unpaired two-tailed Student’s t test or one-way analysis of variance with Bonferroni *t* post-test, depending on the conditions and group number. A *P* value of less than 0.05 was considered to indicate statistical significance.

## Results

### The expression of lncRNA *RPPH1* was higher in breast cancer

**Fig. 1:**
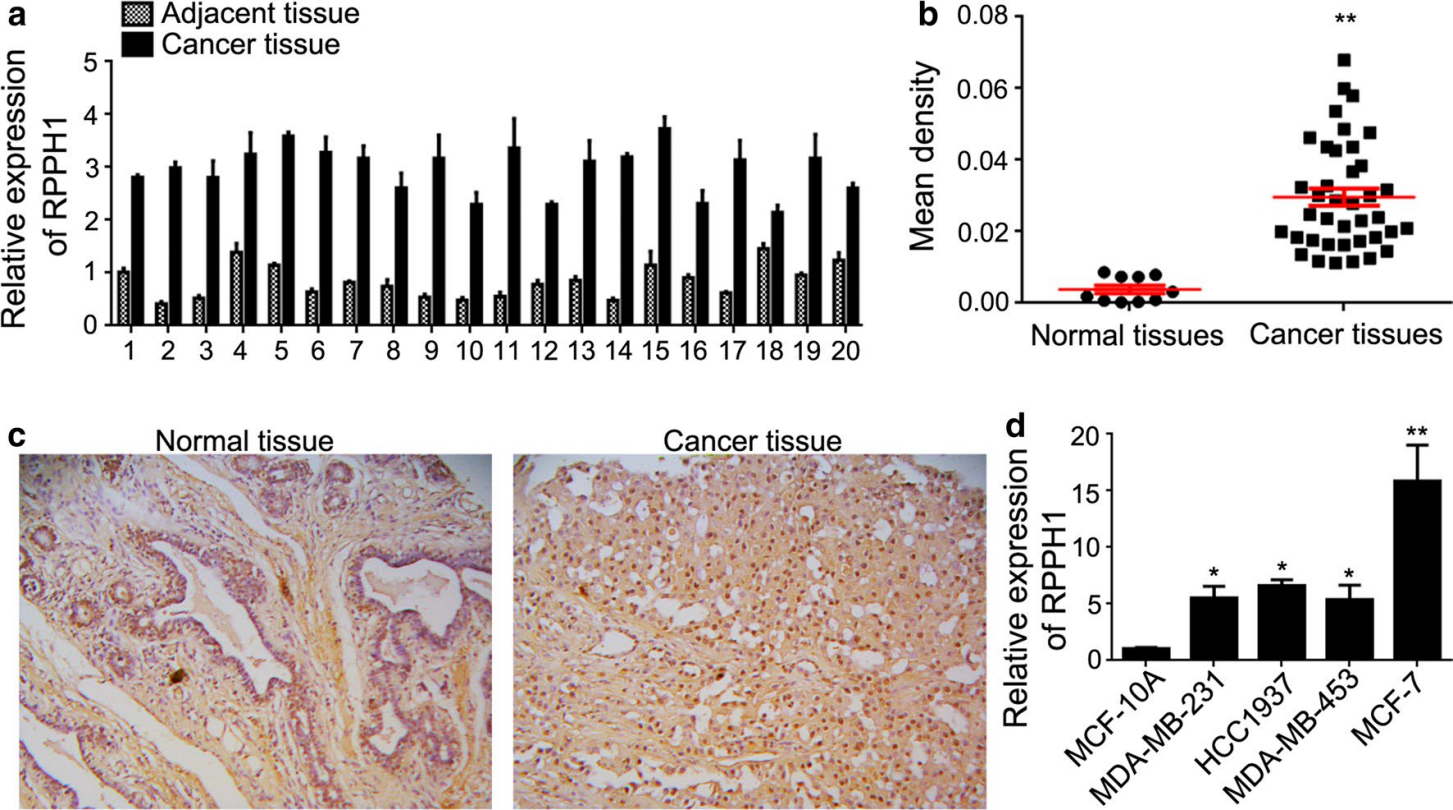
The expression of lncRNA *RPPH1* is upregulated in breast cancer tissues. **a** In the 20 paired clinical samples, the mRNA level of lncRNA *RPPH1* is increased in cancer tissues when compared with the adjacent tissues. **b** ISH assay shows that the level of lncRNA *RPPH1* is relatively high in the cancer sites. **c** Typical ISH assay images show that the expression of lncRNA *RPPH1* is relatively high in sections containing the cancer-site; the brown colour represents positive staining and the blue colour represents the nucleus. **d** The expression lncRNA *RPPH1* in breast cancer cell lines; n = 3, *P < 0.05, **P < 0.01 vs. normal tissues or HBL-100

To explore the potential role of lncRNA *RPPH1* in breast cancer, 20 pairs of cancer-site tissues with adjacent normal tissues were collected from clinical operations. The expression of the lncRNA *RPPH1* gene was significantly increased in cancer sites when compared with adjacent sites, as demonstrated by qPCR (Fig. 1a), and in the breast tissue chip, as demonstrated by ISH (Fig. 1b). Moreover, the expression of lncRNA *RPPH1* in the cytoplasm and nucleus of cancer tissue cells was higher than that in normal tissues (Fig. 1c). Among the cell lines of MDA-MB-231, HCC-1937, MDA-MB-453 and MCF-7, lncRNA *RPPH1* expression was the highest in MCF-7 and the lowest in MDA-MB-231 (Fig. 1d). Therefore, MCF-7 and MDA-MB-231 were chosen for further study.

### LncRNA *RPPH1* increased cell proliferation and promoted cell cycle in breast cancer cells

**Fig. 2:**
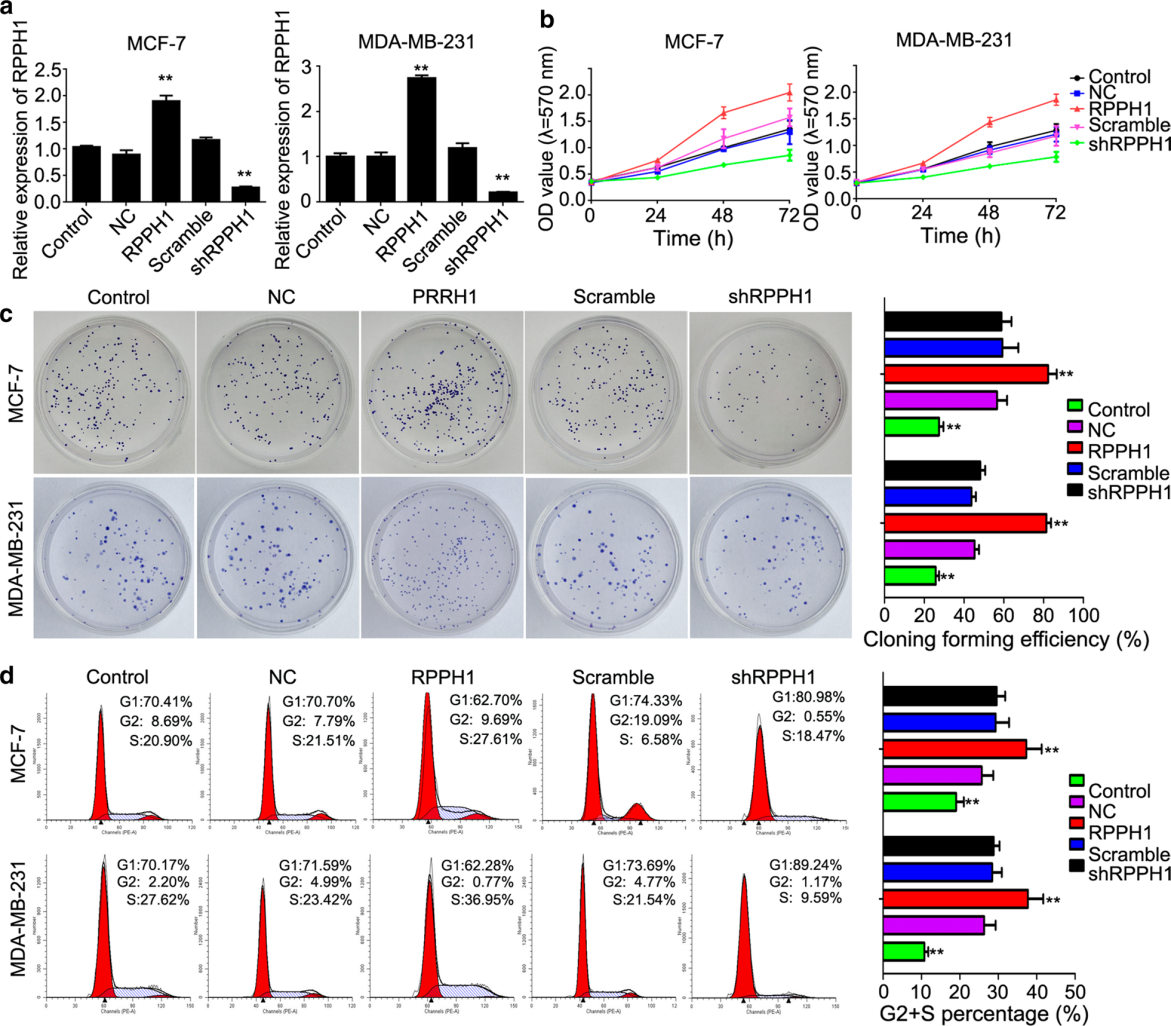
LncRNA *RPPH1* regulates cell proliferation and the cell cycle. **a** qPCR detects successful establishment of the *RPPH1* overexpression and knockdown cell models. **b** The MTT assay shows that cell proliferation is enhanced in the *RPPH1* overexpression model and inhibited in the *RPPH1* knockdown model. **c** The capability of clone formation is increased in the *RPPH1* group and decreased in the sh*RPPH1* group. **d** Flow cytometer shows that the cell cycle is promoted in the *RPPH1* group and arrested in the sh*RPPH1* group; n = 3, *P < 0.05, **P < 0.01 vs. control

Cell models of lncRNA *RPPH1* overexpression and knockdown, which were named *RPPH1* and sh*RPPH1*, respectively, were established by transfecting pcDNA3.1-oe*RPPH1* and pRNAT-U6.1/Neo-sh*RPPH1* vectors, respectively. The following results were similar between the cell lines MDA-MB-231 and MCF-7. First, qPCR detected a dramatically higher gene expression of lncRNA *RPPH1* in the *RPPH1* group compared with the sh*RPPH1* group (Fig. 2a), indicating successful construction of the lncRNA *RPPH1* overexpression and knockdown models. Further evaluation by the MTT assay of lncRNA *RPPH1* in the breast cancer cell lines showed that cell proliferation was increased in the overexpressed vector, but suppressed in the knockdown model (Fig. 2b). In both cell lines, the rate of clone formation in *RPPH1* was increased when compared with the control group and almost doubled when compared with the sh*RPPH1* group (Fig. 2c). Lastly, flow cytometer assay showed that the S+G2 phase of the cell cycle decreased in the *RPPH1* group, but increased in the sh*RPPH1* group (Fig. 2d); indicating that *RPPH1* regulates the cell cycle mainly in the G1 phase.

### *RPPH1* inhibited tumour formation

**Fig. 3:**
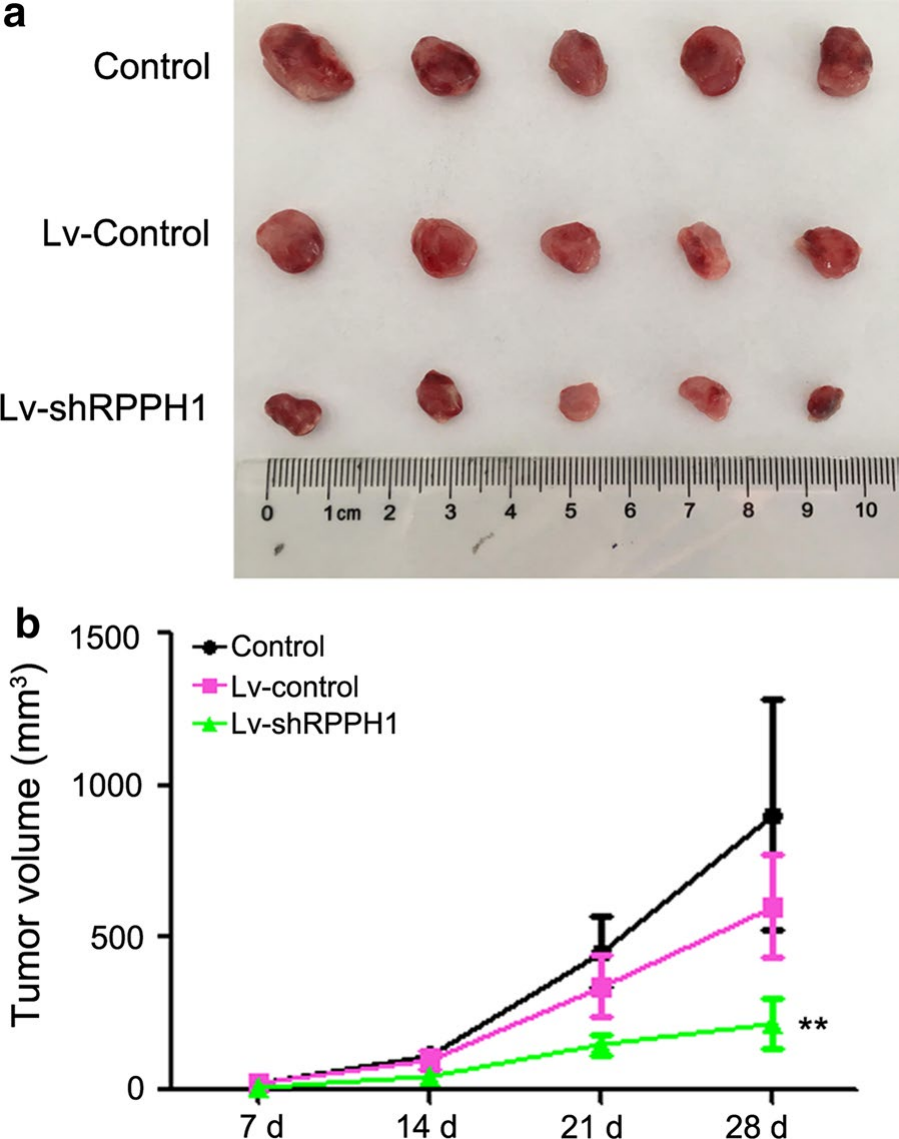
*RPPH1* influences tumour growth in nude mice. **a** Lentivirus-interferenced LncRNA *RPPH1* inhibits tumour growth in nude mice. **b** The tumour growth curve is shown; n = 5, **P < 0.01 vs. control

Further evaluation of the effect of *RPPH1* in solid tumour formation in nude mice showed that lentivirus-mediated interference of sh*RPPH*-*1* lncRNA resulted to obvious changes in tumour size in vivo (Fig. 3a). Statistical evaluation of tumour volume showed that the interactions of *RPPH1* can decrease the size of breast cancer in nude mice (Fig. 3b).

### miR-122 is a target gene of lncRNA *RPPH1*

**Fig. 4:**
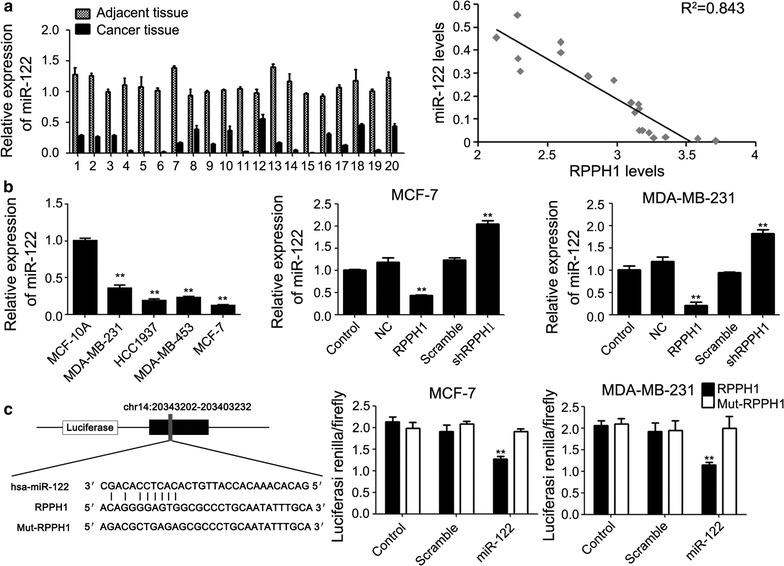
*miR*-*122* is a target gene of lncRNA *RPPH1*. **a** In the 20 paired clinical samples, miR-122 expression in cancer tissues is lower than that in adjacent tissues and the relativity expression rate between miR-122 and *RPPH1*. **b** In the breast cancer cell lines and models of *RPPH1* overexpression and knockdown, the expression of miR-122 is opposite to that of *RPPH1*. **c** The binding area and results of expression in the dual-luciferase assay; n = 3, **P < 0.01 vs. control

Prediction of the possible target binding gene site of lncRNA *RPPH1* using the online software microRNA.org showed that *RPPH1* had a target binding capacity with miR-122, which has complementary pairing of eight bases (Fig. 4c). As shown in Fig. 4a, qPCR to detect the expression level of target microRNA in the 20 cases of breast cancer and the corresponding adjacent tissues showed that the expression of miR-122 was higher in cancer tissues than in the adjacent tissues. Correlation analysis revealed a high value of R^2^ = 0.8431, indicating that miR-122 may possibly be the target binding gene of *RPPH1* in breast cancer. The level of miR-122 expression had an almost opposite trend with the level of *RPPH1* expression in the four kinds of breast cancer cell lines, as well as in the *RPPH1* overexpression and knockdown models (Figs. 1a, 4b). Then, we used dual-luciferase experiment to verify the targeting effect relationship. The luciferase activity significantly decreased in the co-transfected *RPPH1*-psi-CHECK2 plasmid and in the miR-122 mimics in MDA-MB-231 and MCF-7 cell samples, compared with the other groups. These results indicated that lncRNA *RPPH1* and miR-122 in MDA-MB-231 and MCF-7 cells had a targeting effect relationship (Fig. 4c).

### *RPPH1* regulated breast cancer via miR-122

**Fig. 5:**
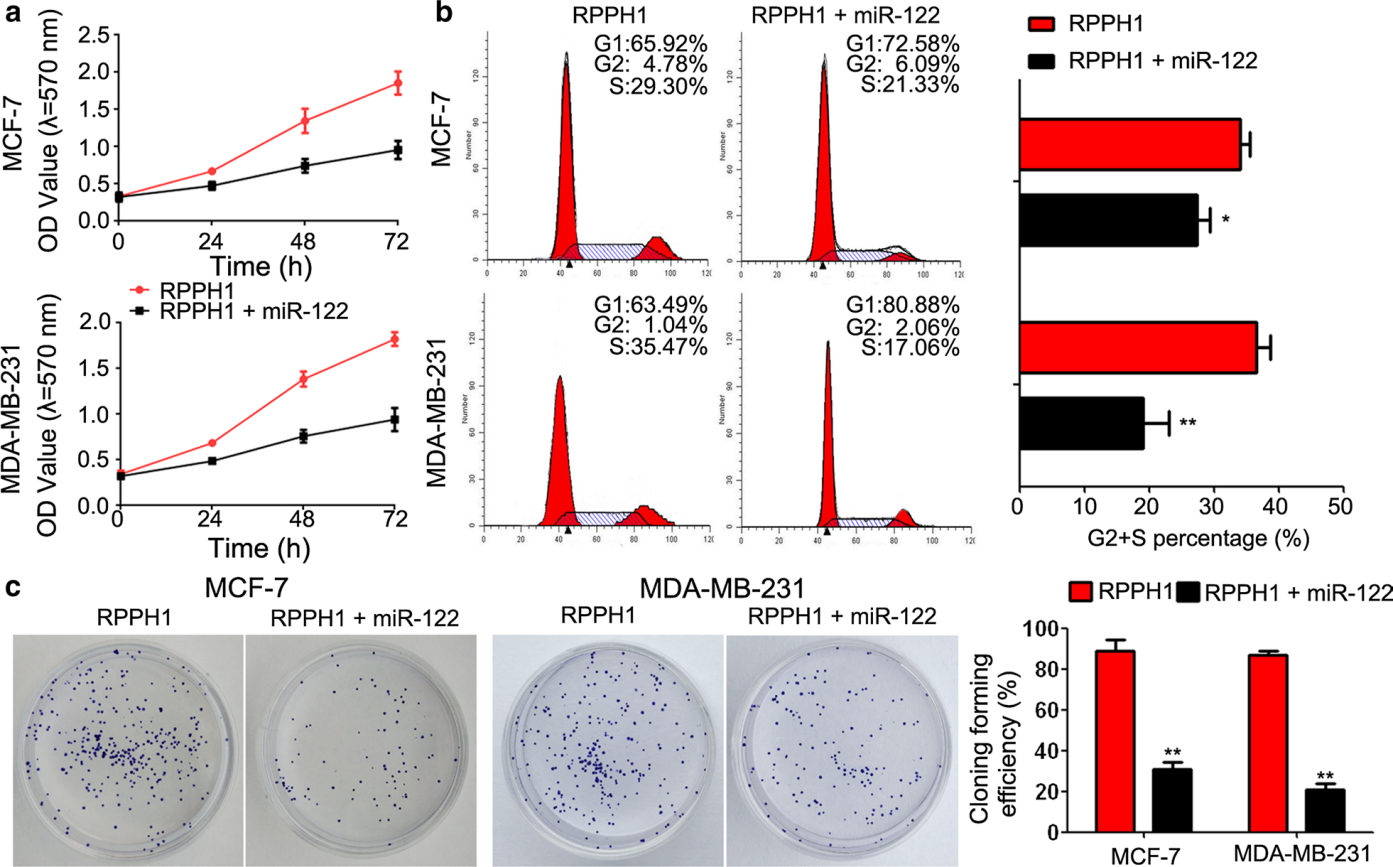
The cell biological characteristics caused by *RPPH1* are reversed by miR-122. Addition of miR-122 mimics to cells with overexpressed *RPPH1*: **a** inhibits cell proliferation, as shown by the MTT assays; **b** suppresses the cell cycle, as shown by flow cytometry and **c** decreases the capability of clone formation; n = 3, *P < 0.05, **P < 0.01 vs. the *RPPH1* group

Because the target gene of *RPPH1* was discovered to be miR-122, we added miR-122 mimics into the *RPPH1* overexpressed model to detect the biological functions of MCF-7 and MDA-MB-231 cells. MTT assay showed that the proliferation of both cells was suppressed in the presence of the miR-122 mimics (Fig. 5a). In addition, the miR-122 mimics reversed the cell cycle promoting ability of the *RPPH1* plasmid (Fig. 5b). Clone formation assay showed that the miR-122 mimics inhibited the capacity for clone formation in the *RPPH1* cell group (Fig. 5c).

### MiR-122 regulated several genes in breast cancer cells

**Fig. 6:**
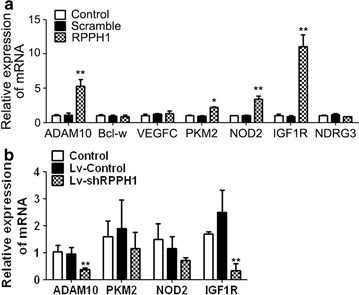
qPCR of the expression of the miR-122 target genes. The expression levels of the target genes of miR-122: **a** in the *RPPH1* overexpression cell model (n = 3), **b** in the *RPPH1* knockdown tumour xenografts (n = 4); *P < 0.05, **P < 0.01 vs. control

MiR-122, which has been reported to have a role in cancers, has diverse target genes; we chose seven of these to explore the downstream genes of miR-122 when *RPPH1* was overexpressed in breast cancer cells. As illustrated in Fig. 6a, the miR-122 target genes *ADAM10*, *PKM2*, *NOD2* and *IGF1R* were highly expressed in the cells of the *RPPH1* overexpression model, whereas the expression of *Bcl*-*w*, *VEGFC* and *NDRG3* had no significant changes. Testing of the RNA expression levels of the four upregulated genes in the tumour xenografts showed that decreased expression of the *ADAM10* and *IGF1R* genes in the *RPPH1* knockdown group (Fig. 6b).

## Discussion

In this study, we first found that lncRNA *RPPH1* was significantly upregulated in breast cancer tissues compared with adjacent normal tissues; these findings corresponded with the result of microarray ISH. In addition, qPCR detected high expression of *RPPH1* in four kinds of breast cancer cell lines. Based on these results, we assumed that *RPPH1* plays an important role in the development of breast cancer and its high expression may help promote tumour deterioration; therefore, *RPPH1* may be a promising prognostic biomarker for breast cancer.

To further explore the functions of *RPPH1* in breast cancer, we speculated that *RPPH1* played a significant role in tumour biology. First, *RPPH1* expression in chosen representative breast cancer cell lines was investigated and compared with that in non-tumour breast cell lines. In agreement with our findings in breast cancer tissues, we found that the MCF-7 and MDA-MB-231 cell lines exhibited high *RPPH1* expression, compared with the HBL-100 cell line. We then determined whether *RPPH1* expression influenced the proliferation of breast cancer cells. Indeed, overexpression of *RPPH1* enhanced cell proliferation and increased clone formation, whereas the knockdown of *RPPH1* significantly inhibited cell proliferation in both MCF-7 and MDA-MB-231 cell lines. Moreover, we demonstrated that the mechanism may be associated with arrest of the G1 phase of the cell cycle, which indicated that *RPPH1* has an ability to regulate the cell cycle. These results revealed that *RPPH1* may affect breast cancer progression by affecting cell proliferation and the cell cycle.

*RPPH1* is a well-known RNA subunit of RNase P, which is responsible for tRNA maturation in all three domains of life [17]. A previous study demonstrated that one of our tested ceRNA pathways, *RPPH1*/miR-330-5p/CDC42, may be involved in the compensatory behaviour of brain neurons to combat synaptic loss during AD pathogenesis [18]. However, the downstream pathway in breast cancer is currently not known. To explore the molecular mechanism through which *RPPH1* contributes to cell proliferation and causes apoptosis in breast cancer cells, we investigated the potential target genes involved in cell proliferation and the cell cycle through microarray analysis. qPCR analysis demonstrated that the miR-122 mRNA levels were decreased in breast cancer cell lines and in cancer-site tissues, compared with the adjacent tissues. Moreover, the expression of miR-122 increased after *RPPH1* knockdown and decreased after *RPPH1* overexpression. Indeed, the dual-luciferase assay demonstrated that *RPPH1* and miR-122 had a relationship of targeted binding. Therefore, we identified miR-122 as a target gene of *RPPH1*.

Further study by addition of miR-122 mimics into the *RPPH1* overexpression model showed that the biological function of *RPPH1* in breast cancer was related to the regulation of miR-122 and that the cell biological functions, including proliferation, cell cycle and clone formation were changed to some extent. In addition, we confirmed that *RPPH1* and miR-122 interacted with each other.

MiR-122, which plays a role in tumour suppression in various cancers, has been investigated extensively. In this study, we focused on the potential effectiveness of miR-122 in breast cancer. Ergun et al. [19] suggested that miR-122-5p was a potential regulator of *ADAM10* and trastuzumab resistance. Sercan Biyun Wang et al. [20] found that miR-122 took the crucial role of a tumour suppressor by targeting *IGF1R* and regulating the PI3K/Akt/mTOR/p70S6K pathway in breast cancer. Another study demonstrated that miR-122 was strongly correlated with the clinical outcomes of breast cancer, including response to neoadjuvant chemotherapy and relapse with metastatic disease. In particular, higher circulating miR-122 levels estimated the occurrence of metastasis in stage II and III breast cancer cases [21]. Based on the results of previous studies, we chose the miR-122 target genes *ADAM10* [19], *Bcl*-*w* [22], *VEGFC* [23], *PKM2* [24], *NOD2* [25], *IGF1R* [20] and *NDRG3* [26] to explore the downstream genes in breast cancer. Based on the qPCR results in the *RPPH1* function cell models, we may infer that *RPPH1* promoted breast cancer cell proliferation and cell cycle progression through down-regulation of micro-RNA 122 and influence the expression of *ADAM10*, *PKM2*, *NOD2* and *IGF1R* genes. The next step should be selecting one of the significant genes to clarify the specific pathway that interacts with *RPPH1*. Finally, investigation of tumour formation in nude mice in this study showed that lentivirus-mediated *RPPH1* lncRNA interference can reduce the size of solid tumours.

## Conclusion

Overall, our results demonstrated that *RPPH1* functions as a tumour promoter and plays an important role in advancing tumorigenesis by targeting miR-122 and may serve as a novel and potential therapeutic, diagnostic or prognostic target in breast cancer.

## Abbreviations

lncRNA: long non-coding RNA
*RPPH1*: ribonuclease P RNA component H1
qPCR: RNA extraction and quantitative real-time polymerase chain reaction
ISH: in-situ hybridisation assay
PBS: phosphate-buffered saline
NCT: neoadjuvant chemotherapy
